# The Transcriptional Response in Human Umbilical Vein Endothelial Cells Exposed to Insulin: A Dynamic Gene Expression Approach

**DOI:** 10.1371/journal.pone.0014390

**Published:** 2010-12-22

**Authors:** Barbara Di Camillo, Tiziana Sanavia, Elisabetta Iori, Vincenzo Bronte, Enrica Roncaglia, Alberto Maran, Angelo Avogaro, Gianna Toffolo, Claudio Cobelli

**Affiliations:** 1 Information Engineering Department, University of Padova, Padova, Italy; 2 Division of Metabolic Diseases, Department of Clinical and Experimental Medicine, University of Padova, Padova, Italy; 3 Istituto Oncologico Veneto (IOV), Istituto Di Ricovero e Cura a Carattere Scientifico, Padova, Italy; 4 Department of Biomedical Sciences, University of Modena and Reggio Emilia, Modena, Italy; 5 BioPharmaNet, Inc., Emilia-Romagna High-Tech Network, Ferrara, Italy; 6 Venetian Institute of Molecular Medicine (VIMM), Padova, Italy; University of Tor Vergata, Italy

## Abstract

**Background:**

In diabetes chronic hyperinsulinemia contributes to the instability of the atherosclerotic plaque and stimulates cellular proliferation through the activation of the MAP kinases, which in turn regulate cellular proliferation. However, it is not known whether insulin itself could increase the transcription of specific genes for cellular proliferation in the endothelium. Hence, the characterization of transcriptional modifications in endothelium is an important step for a better understanding of the mechanism of insulin action and the relationship between endothelial cell dysfunction and insulin resistance.

**Methodology and principal findings:**

The transcriptional response of endothelial cells in the 440 minutes following insulin stimulation was monitored using microarrays and compared to a control condition. About 1700 genes were selected as differentially expressed based on their treated minus control profile, thus allowing the detection of even small but systematic changes in gene expression. Genes were clustered in 7 groups according to their time expression profile and classified into 15 functional categories that can support the biological effects of insulin, based on Gene Ontology enrichment analysis. In terms of endothelial function, the most prominent processes affected were NADH dehydrogenase activity, N-terminal myristoylation domain binding, nitric-oxide synthase regulator activity and growth factor binding. Pathway-based enrichment analysis revealed “Electron Transport Chain” significantly enriched.

Results were validated on genes belonging to “Electron Transport Chain” pathway, using quantitative RT-PCR.

**Conclusions:**

As far as we know, this is the first systematic study in the literature monitoring transcriptional response to insulin in endothelial cells, in a time series microarray experiment. Since chronic hyperinsulinemia contributes to the instability of the atherosclerotic plaque and stimulates cellular proliferation, some of the genes identified in the present work are potential novel candidates in diabetes complications related to endothelial dysfunction.

## Introduction

Diabetic patients die because of the long term chronic complications, namely cardiovascular macroangiopathy, nephropathy, and neuropathy due to the harmful effects of prolonged hyperglycemia in these tissues. From a pathophysiological standpoint, insulin-resistance, a typical metabolic condition in Type 2 diabetic patients initially induces a compensatory hyperinsulinemia, which carries on a proliferative effect among the cellular component of the vascular wall. Chronically elevated insulin concentrations may promote vascular lesion formation; in patients with insulin resistance, such as those with metabolic syndrome, there is an increased risk of cardiovascular disease [1], [2]. Further, hyperinsulinemia contributes to for the instability of the atherosclerotic plaque: it increases the active forms of matrixmetalloproteinases (MMP)-2, MMP-9, and membrane type 1-MMP and the gelatinolytic activity of MMP-2 [3]. Furthermore, insulin may exerts a vasodilator action mediated by phosphatidylinositol 3-kinase (PI3K)-dependent signaling pathways that stimulates the production of nitric oxide from vascular endothelium. In states of insulin resistance, shared glucotoxicity, lipotoxicity, and inflammation selectively impair PI3K-dependent insulin signaling pathways: this contributes to the reciprocal relationships between insulin resistance and endothelial dysfunction [4]. In addition, insulin exerts a plethora of other effects such as the suppression of nuclear factor (NF)-κB, intracellular adhesion molecule (ICAM)-1, monocyte chemoattractive protein (MCP)-1, and of NADPH oxidase [5].

Nonetheless, it is unknown whether insulin itself could increase the transcription of specific genes in the endothelial cells. This is biologically relevant since endothelium itself has a powerful regulatory effect on the underlying vascular smooth muscular cells [6], [7]. Hence, the characterization of the global pattern of transcriptional modifications in the endothelium is important for better understanding the mechanism of action of insulin. The development of microarray technology represents a powerful tool for characterizing such large-scale changes in transcript levels. For example, this methodology was applied to investigate the effects of intensive insulin treatment for 10 days on the mRNA profile in skeletal muscle of type 2 diabetic patients [8]. With a similar methodology, it has been shown that insulin directly modulates the mRNA levels of about 800 genes induced by 3 hours of euglycemic hyperinsulinemic clamp in the vastus lateralis muscle of healthy lean subjects [9]. More recently, it has been shown that insulin is able to regulate different processes within the placenta at different gestational stages, using a global microarray analysis of primary trophoblasts [10]. In pre/post stimulus studies in which the transcriptional response is monitored at one specific time instant after a prolonged insulin exposure, genes showing a transient response followed by a return to the pre-stimulus expression or a systematic, but small in magnitude, change in the expression, are likely to be missed [11]. On the opposite, monitoring the dynamic response using more than one time samples after the stimulus allows detecting these genes as differentially expressed and provides a description of the transcriptional expression patterns of the response. Transient behavior might be characteristic, and, if common to a number of genes associated to the same functional group, might give insight into the function performed by the gene circuitry. The aim of the present work is to exploit the potential of a dynamic study to investigate the dynamic transcriptional response of endothelial cells following insulin stimulation. As far as we know, this has not been previously addressed in the literature for endothelial cells stimulated with insulin.

To distinguish between insulin effect and other processes that take place in the cell simultaneously, but are not induced or inhibited by insulin, treated cells were compared with control cells. Experiments were carried out on human umbilical vein endothelial cells (HUVECs). As far as we know, this is the first systematic study in the literature monitoring transcriptional response to insulin in endothelial cells, in a time series microarray experiment.

## Materials and Methods

### Cell Cultures

Human umbilical vein endothelial cells (HUVECs) were obtained from Promocell (PromoCell, Heidelberg, Germany) from a single donor. These cells were certified to be free from mycoplasma contamination and tested negative for HIV, HBV and HCV virus infections or contamination by PCR results. Cells were cultured in Endothelial Cell Growth Medium (PromoCell) which is a modified MCDB 131 medium, supplemented with 10% fetal bovine serum (FBS) (Sigma Aldrich, Saint Louis, USA), 0.02% Supplement Mix/ Endothelial Cell Growth Medium (PromoCell), 100 U/ml penicillin and 100 µg/ml streptomycin (Sigma Aldrich). All cells were maintained in a humidified 5% CO2 incubator at 37°C, and the medium was replaced every 2 days until confluence. All the experiments reported in this paper were carried out on cells between 4th and 5th passage.

### Experimental design

HUVECs were seeded onto six well plates at a density of 2×10^5^ per well and cultured as described above. At the second day, cells were incubated overnight with 1% FBS medium, without growth factors. The day of the experiment, 1 ml of quiescent medium, in absence or presence of 1 mU/ml (7 nM) insulin (Calbiochem-Inalco Spa), was added to each well. Cells of three wells were pooled and used for each time point. Samples were collected at times 0, 40, 100, 200, 340, 440 min. Time 0 was cultured and harvested in duplicate so to have a complete experimental replicate of time 0 sample. HUVECs were harvested at the designated time points, and RNA was extracted and quantified for Microarray analysis (Affymetrix measurement) The medium with insulin was collected and stored at −20°C for the insulin detection (Insulin Myria, Technogenetics), while the medium without insulin was discharged. In order to validate the Affymetrix measurements, a quantitative real-time polymerase chain reaction was performed. The experimental design described above was repeated. Samples were collected at time 0, 30, 60, 120, 180, 240, 300, 360, 420, 480 min. All the experiments were carried out in duplicate.

### RNA extraction

Cells were washed with phosphate buffered saline (PBS). Total RNA was extracted using a commercially available kit (TRIzol Reagent, Invitrogen) and stored at −80°C. The samples were further purified using RNeasy mini kit (Qiagen, Milan, Italy) following manufacturer's recommendations. The integrity of RNA was systematically checked by use of the lab-on-chip technology in an Agilent Bioanalyzer 2100 with the RNA6000 Nano Assay (Agilent Technologies, Palo Alto, CA). Furthermore the purity was determined by spectrophotometric readings at 260/280/230 nm.

### Affymetrix Measurements

Total RNAs were purified using an RNeasy Protect Mini Kit from Qiagen. The quality and quantity of total RNA was measured using the Agilent test on a Bioanalyzer (Agilent Technologies, Palo Alto, CA). Gene transcript profiles in both control and treated cultures were studied by high-density oligonucleotide microarrays Human Genome U133 Plus 2.0 GeneChip (Affymetrix, Santa Clara, CA). Sample labeling, hybridization of test array, and hybridization of full-size arrays were performed using protocols described in the Affymetrix GeneChip expression analysis technical manual.

### Affymetrix Data Analysis

**Figure 1:**
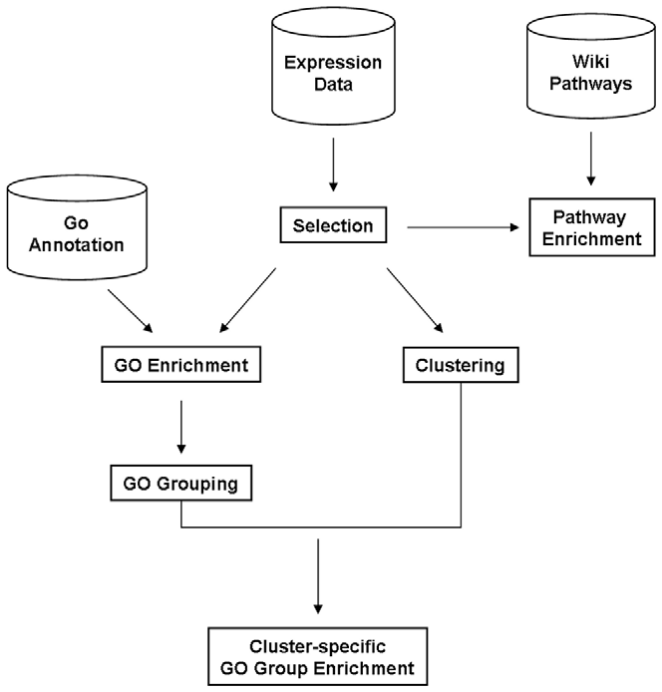
Pre-processed Affymetrix data analysis pipeline. A selection method was applied to identify differentially expressed genes. Selected genes were clustered according to their time expression profile; significantly associated pathways and GO terms were identified through enrichment analysis. The enriched GO terms were grouped into different functional categories. For each cluster and for each GO category, GO enrichment based on Fisher's Exact Test was calculated.

Image quantification was performed using GeneChip (Affymetrix, SantaClara, CA) scanner and software. Preprocessing steps such as background subtraction, probe cell normalization and expression level calculations, were performed using quantile normalization and Robust Microarray Analysis (RMA) software [12]. High-level data analysis was carried out in a pipeline as shown in Figure 1.

#### Selection

Differentially expressed genes were selected using the method described in [11] that calculates the area of the region bounded by the treated-minus-control expression profile and assigns a p-value to each gene by evaluating the significance of this area against the null hypothesis. The two replicates available at time zero were used to derive the experimental error distribution at different intensity expression values and, consequently, the null hypothesis distribution of the area bounded by the treated-minus-control expression profile. As already shown in [11], the method, implemented for data poor conditions, is quite robust to random oscillation, and help diminishing both false positive and false negative rates. In order to account for multiple testing, the significance level was corrected according to a false discovery rate (FDR), i.e. the number of false positives divided by the number of selected genes, of 0.05.

#### GO and pathway enrichment

Genes were annotated according to molecular functions of Gene Ontology (GO) database [13], using NetAffx database (http://www.affymetrix.com/analysis/index.affx). Enrichment analysis was performed based on a strategy similar to the “elim” method described in [14]. GO terms (related to different molecular functions) were grouped into levels according to the percentages of selected genes: namely, level 1 corresponds to GO terms with at least 98%–100% of their annotations selected, level 2 to the range 96%–98%, etc. Starting from level 1, the algorithm visited each level and for each GO term performed Fisher's Exact Test that assigns a p-value representing the probability that the observed number of selected genes annotated to the GO term could have resulted from random sampling. If in the visited level a GO term has a p-value below a significance level α, then the corresponding genes were removed from the annotation of GO terms having lower percentages, in order to penalize their p-value. In this way, the number of enriched GO terms was kept low, still maintaining a high significance level. Since this test was applied to a large number of GO terms, the significance level α for the calculated p-values was empirically set to 0.0025.

To identify the most enriched pathways, selected genes were also annotated to WikiPathways (http://www.wikipathways.org) using NetAffx database. Enrichment analysis of pathways was performed using Fisher's Exact Test.

#### GO grouping

To obtain a more synthetic annotation, the enriched nodes directly connected by a path in the GO graph were grouped together in the same functional cluster. Each GO group, thus characterized by an isolated sub-graph of siblings or ancestors terms, was labeled with the most general of these terms.

#### Clustering

To identify the main temporal expression patterns in response to insulin stimulus, treated-minus-control expression profiles of selected genes were clustered using K means clustering based on Pearson correlation. The number K of clusters was set to 7.

#### Cluster-specific GO group enrichment

For each cluster and for each GO group defined above, GO enrichment based on Fisher's Exact Test was calculated separately, so that the resulting p-value represents the probability that the observed numbers of selected genes belonging to the cluster and annotated with the GO group have resulted from random sampling. GO groups with p-value<0.05 were considered as significantly enriched for the cluster.

### Validation via qRT-PCR

Affymetrix data validation was performed using qRT-PCR, by monitoring a subset of 32 genes belonging to the electron transport chain pathway, of which 21 selected and 11 not selected as differentially expressed using Affymetrix data (see results section). HUVECs for this experiment were obtained from a second donor (Promocell). Real-Time PCR Analysis of Gene Expression. cDNAs were reverse transcribed from total RNA samples (100 ng) using the High Capacity cDNA Reverse Transcription Kit (LifeTechnologies-Applied Biosystems, Foster City, CA, USA). TaqMan PCRs were carried out onto custom TaqMan low-density arrays by means of the ABI PRISM 7900 HT Sequence Detection Systems (all from Life Technologies-AppliedBiosystems). Statistical analyses were obtained using the 2-Delta-delta-Cycle Threshold values (Delta-delta-CTs) method, with the Threshold determined automatically by means of SDS software (Life Technologies-Applied Biosystems). To normalize data, CT was calculated for each detector by using the median of CTs in all samples as calibrator. The relative quantity (RQ) of each mRNA was calculated as 2^−DDCT^. Differentially expressed genes were selected by applying the method described in [11], using a significance level alpha equal to 0.05 on the detected false discovery rate p-values.

The overlap in the lists of differentially expressed genes selected by Affymetrix and qRT-PCR was quantified by means of a contingency table, while the dynamic patterns of expression were compared in terms of up and down regulation.

## Results

### Affymetrix Data Analysis

#### Selection

2326 probe-sets, associated to 1715 genes, were selected as differentially expressed in the cells exposed to insulin.

#### GO and pathway enrichment

Functional annotations of selected genes indicate that 26 molecular functions are significantly affected by insulin (Table 1). In terms of endothelial function, the most prominent processes affected were oxidoreductase activity, acting on NADH or NADPH, quinone or similar compound as acceptor, NADH dehydrogenase activity, NADH dehydrogenase (ubiquinone) activity, NADH dehydrogenase (quinone) activity, cell adhesion molecule binding, protein transporter activity, N-terminal myristoylation domain binding, nitric-oxide synthase regulator activity, and growth factor binding.

**Table 1:** Enriched Gene Ontology molecular functions.

GOID	TERM	Annotated	Selected	p-value
GO:0005515	protein binding	8020	913	1.03E-13
GO:0003723	RNA binding	835	131	5.40E-10
GO:0016462	pyrophosphatase activity	785	102	7.81E-07
GO:0016818	hydrolase activity, acting on acid anhydrides, in phosphorus-containing anhydrides	788	102	9.51E-07
GO:0016817	hydrolase activity, acting on acid anhydrides	796	102	1.59E-06
GO:0016655	oxidoreductase activity, acting on NADH or NADPH, quinone or similar compound as acceptor	57	18	1.62E-06
GO:0003954	NADH dehydrogenase activity	52	16	8.87E-06
GO:0008137	NADH dehydrogenase (ubiquinone) activity	52	16	8.87E-06
GO:0050136	NADH dehydrogenase (quinone) activity	52	16	8.87E-06
GO:0017111	nucleoside-triphosphatase activity	755	95	8.96E-06
GO:0003779	actin binding	341	60	5.87E-05
GO:0051015	actin filament binding	44	14	1.99E-04
GO:0050839	cell adhesion molecule binding	25	9	2.30E-04
GO:0008565	protein transporter activity	97	20	2.94E-04
GO:0008353	RNA polymerase subunit kinase activity	5	4	3.36E-04
GO:0003755	peptidyl-prolyl cis-trans isomerase activity	57	14	3.89E-04
GO:0016859	cis-trans isomerase activity	58	14	4.72E-04
GO:0003743	translation initiation factor activity	74	16	6.66E-04
GO:0031997	N-terminal myristoylation domain binding	3	3	7.92E-04
GO:0004661	protein geranylgeranyltransferase activity	6	4	9.25E-04
GO:0030235	nitric-oxide synthase regulator activity	6	4	9.25E-04
GO:0043566	structure-specific DNA binding	146	25	1.06E-03
GO:0003714	transcription corepressor activity	137	23	1.08E-03
GO:0016303	1-phosphatidylinositol-3-kinase activity	11	5	1.86E-03
GO:0019838	growth factor binding	98	18	1.95E-03
GO:0051082	unfolded protein binding	120	24	2.46E-03

For each enriched GO term the GOID and the corresponding name are shown, with the number of genes annotated in the GO database (Annotated), the number of genes selected as differentially expressed annotated in the GO database (Selected) and the p-value evaluated as described in Methods.

Enrichment analysis revealed one significantly enriched pathways: “Electron Transport Chain”, containing 28 of the selected genes on a total of 118 annotated genes.

#### GO grouping

**Figure 2:**
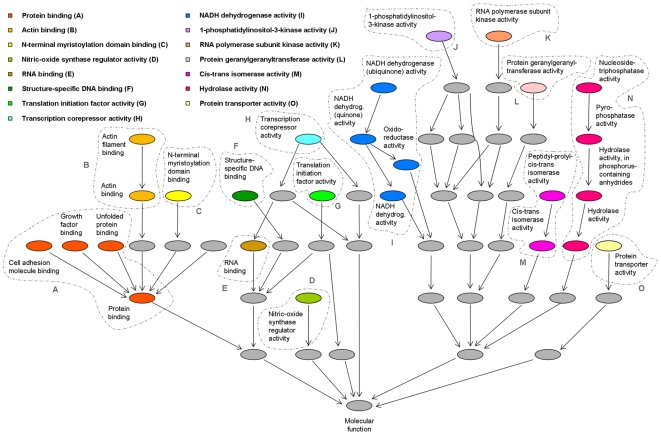
GO graph of enriched molecular function terms. The paths of GO enriched terms are displayed; nodes directly connected by a path in the GO graph were grouped together into 15 GO main annotation groups (denoted by capital letters).

The GO sub-graph containing all the paths from the 26 enriched GO terms to the root is depicted in Figure 2, where enriched GO terms are grouped together into 15 main GO groups, according to the rules explained in Methods; namely, Protein binding, Actin binding, N-terminal myristoylation domain binding, Nitric-oxide synthase regulator activity, RNA binding, Structure-specific DNA binding, Translation initiation factor activity, Transcription corepressor activity, NADH dehydrogenase activity, 1-phosphatidylinositol-3-kinase activity, RNA polymerase subunit kinase activity, Protein geranylgeranyltransferase activity, Cis-trans isomerase activity, Hydrolase activity, Protein transporter activity. Ten out of fifteen groups correspond to isolated nodes in the GO database.

#### Clustering and cluster-specific GO group enrichment

**Figure 3:**
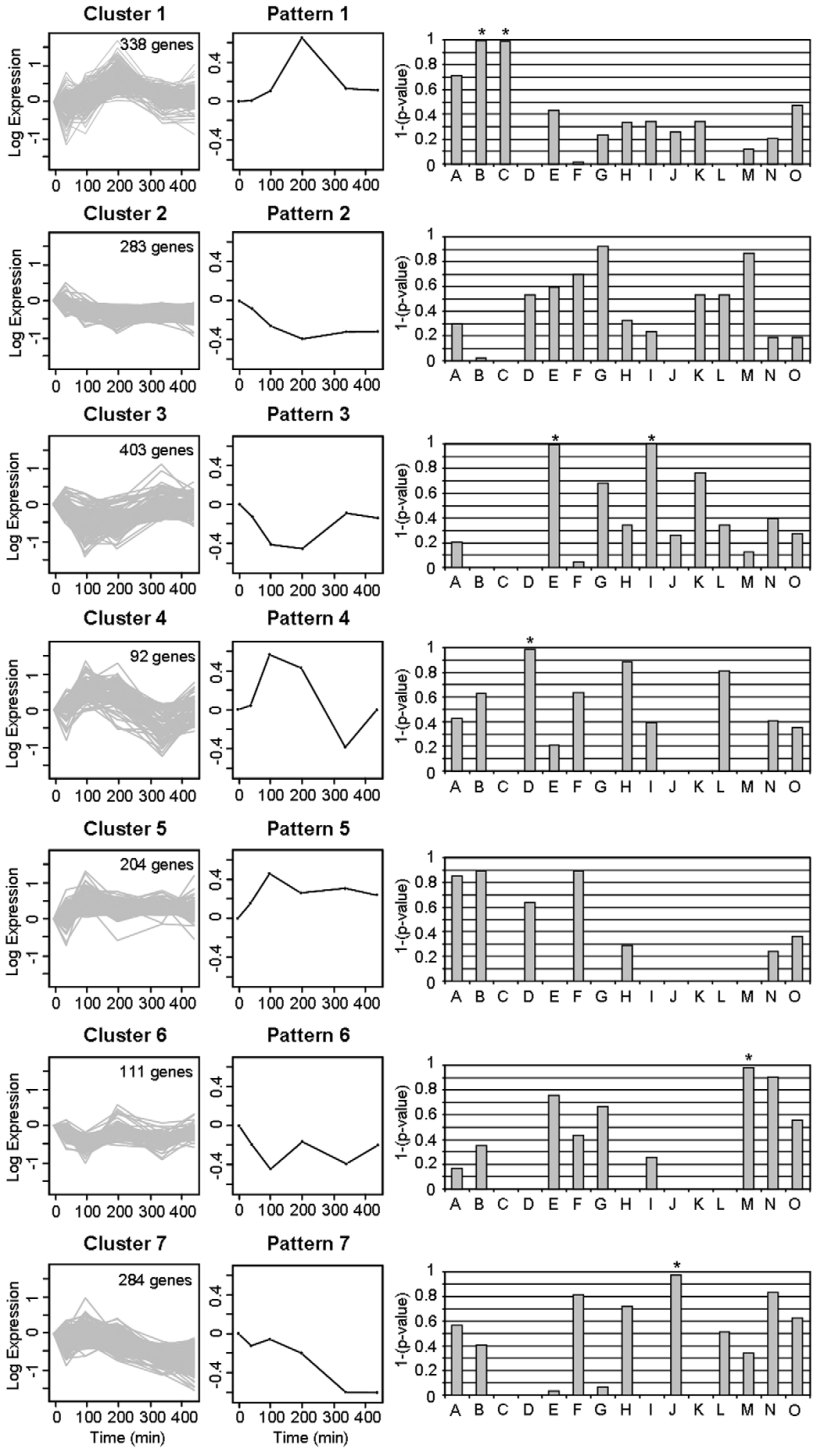
Cluster-specific GO group enrichment. Left panels show temporal expression profiles (treated minus control) of genes belonging to each cluster, identified by K-means algorithm; middle panels represent the corresponding average temporal patterns; right panels show significance of the enrichment for each GO category (identified by capital letters) in each cluster, as 1 minus p-value. A star indicates significant GO groups (with p-value < 0.05).

Seven main temporal patterns in response to insulin stimulus were identified for treated-minus-control expression profiles as shown in Figure 3 (left panels), together with the number of genes correlated to each pattern. For each cluster the specific enrichments in the 15 different GO groups was expressed as (1−p-value), as shown in Figure 3 (right panels), so that a value close to 1 indicates an elevated significance level.

Cluster 1, characterized by a peak of the expression level at time 200 min, is significantly associated with GO groups B (actin binding) and C (N-terminal myristoylation domain binding), in which three genes coding for calmodulin are annotated.

Cluster 2 shows initial down-regulation followed by a plateau; even if genes belonging to GO groups G (translation initiation factor activity) and M (cis-trans isomerase activity) are highly represented in this cluster, it does not reach significance for any GO group.

Cluster 3 shows an initial decrease in the expression level and a return to a pre-stimulus expression, significant in GO groups E (RNA binding) and I (NADH dehydrogenase activity).

Cluster 4 shows a transient rise of expression level, followed by a decrease, significant in GO group D (nitric oxide synthase regulator activity).

Cluster 5 shows up-regulation at time 100 min, followed by a plateau; even if genes belonging to GO group A (protein binding), B (actin binding) and F (structure-specific DNA binding) are highly represented in this cluster, it does not reach significance for any GO group.

Cluster 6 shows down regulation of expression at time 100 min and 340 min, significantly associated with GO group M (cis-trans isomerase activity).

Cluster 7 shows consistent down-regulation, significant in GO group J (1-phosphatidylinositol-3-kinase activity).

Table S1 contains an Excel file with 15 worksheets showing the selected genes belonging to each GO group. In the file, for each group, the list of genes, their symbols, titles, chromosome locations, expression clusters and FDR p-values obtained by selection method are shown. To give a more complete and easy to access information about the direction and magnitude of change, for each time point significant changes in gene expression were identified based on a model of the error obtained using the two available replicates at time 0 [15]. Since the purpose here is to indicate the sign and the magnitude of the change and not to select genes, we fixed the threshold so to minimize the sum of false positive and false negative rates as explained in [15]. The direction and the magnitude of the changes are indicated only when they result significant. Complete time-series expression data of each gene are available in GEO database (GSE21989).

### Validation via qRT-PCR

**Figure 4:**
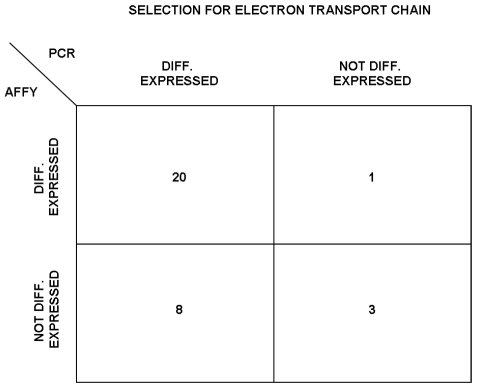
Comparison between Affymetrix and qRT-PCR. Contingency table that compares the lists of selected/not selected genes belonging to Electron Transport Chain pathway for both Affymetrix and qRT-PCR experiments. 21 of the 32 genes monitored by qRT-PCR were selected as differentially expressed from Affymetrix measurements, while 28 were selected from qRT-PCR measurements. In particular, 20 of the 21 genes selected as differentially expressed by using Affymetrix chips are also selected by qRT-PCR. However, 8 genes selected using qRT-PCR are not selected using Affymetrix chips, probably due to the better precision of qRT-PCR technology to measure low RNA concentration with respect to Affymetrix chips.

To validate Affymetrix measurements, 32 genes related to “Electron Transport Chain” pathway were monitored using qRT-PCR, as explained in Methods. As shown in Table 2, 21 of these genes were selected as differentially expressed from Affymetrix measurements, while 28 were selected from qRT-PCR measurements. The overall overlap (Figure 4) is 72%, indicating a good agreement between the two techniques. In particular, 20 of the 21 genes selected as differentially expressed by using Affymetrix chip are also selected by qRT-PCR, showing an overlap of 95%. However, 8 genes selected using qRT-PCR are not selected using Affymetrix chips, probably due to the better precision of qRT-PCR technology to measure low RNA concentration with respect to Affymetrix chips.

**Table 2:** Genes monitored by qRT-PCR.

Gene Symbol	Gene Name	Selected qRT-PCR	Selected Affy	GOgroup	Cluster
ATP5A1	ATP synthase, F1 complex, alpha subunit 1	Yes	No	A	---
ATP5C1	ATP synthase, F1 complex, gamma polypeptide 1	Yes	Yes	N	3
ATPIF1	ATP synthase, F0 complex, subunit B1	Yes	No	A	---
ATP5F1	ATP synthase, F0 complex, subunit B1	Yes	Yes	A,N	2
ATP5G1	ATP synthase, F0 complex, subunit C1	No	No	---	---
ATP5I	ATP synthase, F0 complex, subunit E	Yes	Yes	N	2
ATP5L	ATP synthase, F0 complex, subunit G	No	No	A	---
ATP5O	ATP synthase, F1 complex, O subunit	Yes	No	---	---
BTF3	basic transcription factor 3	Yes	Yes	A	2
CYCS	cytochrome c, somatic	Yes	Yes	A	2
COX17	cytochrome c oxidase assembly homolog	Yes	Yes	---	3
COX6B1	cytochrome c oxidase subunit Vib	Yes	Yes	---	3
COX5B	cytochrome c oxidase subunit Vb	Yes	Yes	---	3
COX7B	cytochrome c oxidase subunit VIIb	Yes	No	---	---
COX7C	cytochrome c oxidase subunit VIIc	Yes	Yes	---	3
NARG1	NMDA receptor regulated 1	Yes	Yes	A	7
NDUFA2	NADH dehydrogenase, alpha, subunit 2	Yes	Yes	I	2
NDUFA3	NADH dehydrogenase, alpha, subunit 3	Yes	Yes	I	2
NDUFA6	NADH dehydrogenase, alpha, subunit 6	Yes	Yes	I	2
NDUFA7	NADH dehydrogenase, alpha, subunit 7	Yes	Yes	I	2
NDUFA12	NADH dehydrogenase, alpha, subunit 12	Yes	Yes	I	2
NDUFB2	NADH dehydrogenase, beta, subunit 2	Yes	Yes	I	2
NDUFB3	NADH dehydrogenase, beta, subunit 3	No	Yes	I	2
NDUFS1	NADH dehydrogenase, Fe-S protein 1	Yes	Yes	A,I	2
NDUFS4	NADH ubiquinone oxidoreductase IP subunit mRNA	Yes	Yes	I	2
NDUFV2	NADH dehydrogenase flavoprotein 2	Yes	No	I	---
SDHA	succinate dehydrogenase, subunit A	Yes	No	---	---
SDHB	succinate dehydrogenase, subunit B	Yes	Yes	A	2
SLC25A6	solute carrier family 25	Yes	No	A	---
UCP2	uncoupling protein 2	No	No	A	---
UQCRB	ubiquinol-cytochrome c reductase binding protein	Yes	Yes	---	3
UQCRC2	ubiquinol-cytochrome c reductase core protein II	Yes	No	A	---

For each of the 32 measured genes, the corresponding symbol and name are shown, with the information on selection results for qRT-PCR (Selected qRT-PCR) and for Affymetrix (Selected Affy). The association with the GO groups and the clusters defined in Figure 3 are also shown.

**Figure 5:**
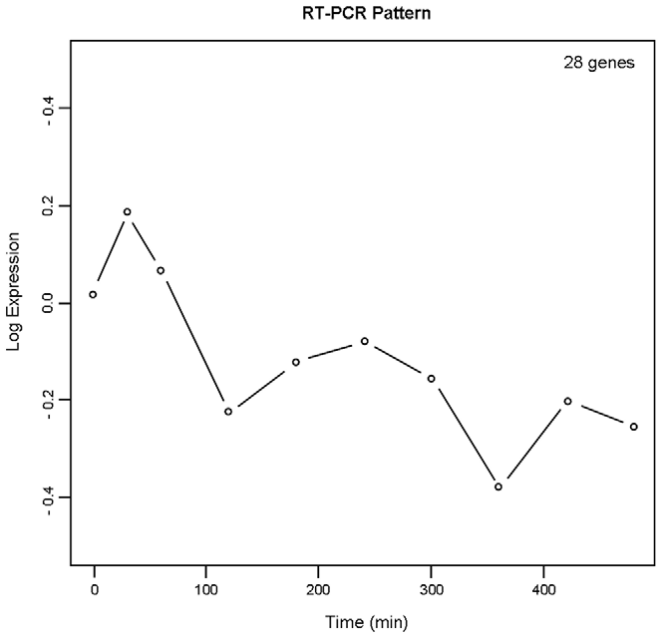
Average profile of the qRT-PCR selected genes. The 28 time series selected with qRT-PCR experiment are represented by the average profile, characterized by an early rise of expression level at time 30 min, followed by an increasing down-regulation.

There is also a good agreement in the time expression profiles of the “Electron Transport Chain” obtained with the two techniques. In fact, the expression profiles of the 21 Affymetrix selected genes are all associated to clusters 2 and 3 (apart from gene NARG1, associated to cluster 7), indicating a prevalent down-regulation of genes belonging to “Electron Transport Chain” pathway. The 28 time series of qRT-PCR selected genes share a similar temporal pattern, characterized by an early rise of expression level at time 30 min, followed by an increasing down-regulation (Figure 5). Since the early response was not monitored by Affymetrix, we can conclude that the two techniques reveal similar response to insulin for genes related to “Electron Transport Chain”.

## Discussion

The objective of this study was to utilize DNA microarray technology to assess the transcriptional response to insulin in endothelial cells, in a time series microarray experiment. These experiments were performed by exposing endothelial cells derived from human umbilical veins to pharmacologic insulin concentrations.

To identify significant transcriptional temporal patterns in endothelial cells treated with insulin and to characterize them from a functional point of view, an ad hoc analysis pipeline was applied to experimental data. In particular: 1) Differentially expressed genes, selected by using a method tailored for gene expression time series in data-poor conditions, were annotated according to Gene Ontology molecular functions; 2) The enriched GO terms were grouped together according to their position in the GO graph in order to obtain a more synthetic annotation and these groups were used to annotate the main temporal expression patterns identified by cluster analysis. This approach selects genes based on their dynamic gene expression profiles, thus detecting even small but systematic changes in gene expression. Then, by integrating cluster analysis and functional annotations, it gives a limited number of non-redundant functional groups.

Application of the analysis pipeline to Affymetrix data demonstrates the followings:

1. Endothelial genomic response is significantly affected by elevated insulin concentrations;
2. These effects are characterized by remarkably different temporal profiles.

Differently from previous works in the literature that monitor gene expression in endothelial cells in static conditions before and after insulin stimuli, we focus here on the dynamic response of endothelial cells to insulin stimulation. This approach allowed us to detect as differentially expressed also genes that respond to insulin with a transient response followed by a return to the baseline condition or with a small, even thought systematic, i.e. sustained in time, change in gene expression. These genes would not have been detected with a pre/post stimulus study of transcriptional response in which only one specific time instant is considered after insulin exposure,. For example, most of the genes in cluster 3 and 4, enriched of genes belonging to RNA binding, NADH dehydrogenase activity and nitric-oxide synthase regulator activity GO groups (Figure 3), would have not been detected by a pre/post stimulus study.

Insulin responsive genes were characterized in terms of dynamic pattern and functional annotation. This is of importance due to the specific contribution of insulin not only to glucose metabolism but also to the vascular homeostasis. It is known that insulin mediates the metabolic-hemodynamic coupling by increasing the microvascular exchange surface perfused within skeletal muscle [16]: this may be relevant in conditions of insulin resistance where, at least in muscle, insulin not only is unable to induce a proper vasodilatation but also the up regulation of genes such hexokinase II, p85aPI3K, and SREBP-1c which are critical for intracellular insulin signaling and glucose transporter recruitment [17]. Consoli and colleagues [18] have also shown that, at endothelial levels, genetic insulin resistance may be postulated, leading to a possible imbalance of prothrombotic and fibrinolytic genes.

Notwithstanding that arterial and venous derived endothelial cells differ from HUVECs for anatomical, functional, and transcriptional identities, we select the latter in our experimental setting [19], [20] since they are commonly employed in experimental protocols which investigate the effects of insulin on endothelial functions [21] and on gene expression [16].

Our data show that insulin exposure is associated in HUVECs with different patterns of differential expression. Cluster 1 (Figure 3) relates to N-terminal myristoylation domain binding a molecular function which indicates the selective interaction with the N-terminus of a protein; binding affinity is altered by myristoylation. On this specific effect only few reports are available. Interestingly, in an experimental model of diabetic rodents, the N-myristoyltransferase in the liver is increased as compared to control whereas in obese rodents is decreased. In the endothelial cells this process may be of importance since it was shown that the eNOS is post-translationally modified by myristoylation of Gly2 [22], [23] and it is important in the interplay between phosphorylation and subcellular localization of eNOS [24].

In cluster 4, we have shown a rise, albeit transitory, of the GO group D which refers to nitric oxide synthase regular activity. As expected, insulin exposure is able to increase NO production by endothelial cells [25]. Nitric oxide synthase gene expression may be regulated at multiple levels: epigenetic, transcriptional, and posttranscriptional processes [26]. As shown by Kuboki and colleagues in cultured bovine aortic endothelial cells, insulin can regulate the expression of eNOS gene, mediated by the activation of PI-3 kinase [27]. This observation has been further replicated [28]. Very little information is available in the literature about the temporal stimulation of nitric oxide gene activation at least in arterial derived endothelial cells. Yet, at least endothelial nitric oxide synthase protein may be characterized by a remarkable temporal expression in rat femoral artery [29]. Our differential expression pattern shows that the GO group “nitric oxide synthase regulator activity” is temporally regulated by insulin stimulation. This observation might be taken with caution since eNOS expression itself is not changed. Furthermore, this temporal expression is detected by incubating the endothelial cells at pharmacologic insulin concentration. It is thus impossible to make any inference about the possible temporal effect in the presence of physiological concentrations of the hormone.

We have also shown that in cluster 7 the 1-phoshatydilinositol-3-kinase activity is negatively expressed. PI3K/Akt is involved in both cytosolic and nuclear signaling in endothelial cell where it regulates vascular homeostasis and angiogenesis [30], [31]. PI3K signaling mediates Akt/PKB phosphorylates eNOS at serine-1177 residue [32]–[34]. Insulin stimulates NO release by activating PI3/Akt signaling [25]. Therefore, it is clear that insulin affects NO production mainly through protein phosphorylation rather than regulating protein gene expression [35]. It is therefore unclear why we observed a negatively differentially expressed PI3K activity and a divergent effect of gene expression of these two pathways, NOS synthase activity and PI3K activity, apparently linked to each other. This effect may be determined obviously by different regulation of their gene expressions by insulin. Therefore, the significance of these findings merits further consideration.

Our data show that insulin exposure significantly reduces the average expression of genes in the electron transport chain (Figure 5), with significant enrichment in GO group I: NADH dehydrogenase activity (cluster 3, Figure 3). These findings emphasize that electron transport chain is significantly regulated by insulin and, probably, negatively regulated by the chronic exposure to elevated concentrations of the hormone. Indeed, it has been shown that insulin not only regulates this pathway [36] but also that, in the presence of insulin resistance, electron transport chain can be deeply altered [37], [38].

In conclusion, the present data demonstrate that insulin affects mRNA levels of about 1700 genes in HUVECs. These genes can be clustered in groups with characteristic time expression profile and classified into functional categories that can support the biological effects of insulin. Microarray data were confirmed by measuring the mRNA levels of a subset of genes using quantitative real-time PCR. An important issue now is to understand how insulin coordinates the expression of all these genes. The identification of common elements in the promoter sequences of group of genes will help the discovery of the transcription factors linking the effect of insulin on multiple genes simultaneously. In addition, since chronic hyperinsulinemia contributes to the instability of the atherosclerotic plaque and stimulates cellular proliferation, some of the genes identified in the present work are potential novel candidates in diabetes complications related to endothelial dysfunction. More focused studies on subsets of genes and on several donors will be objective of future studies.

## Supporting Information

Table S1 Selected genes. Table S1 contains an Excel file with 15 worksheets showing the selected genes belonging to each GO group. In the file, for each group, the list of genes, their symbols, titles, chromosome locations, expression clusters and FDR p-values obtained by selection method are shown.
(0.40 MB XLS)
